# Validation of dynamic three-dimensional whole heart magnetic resonance myocardial perfusion imaging at 3.0 Tesla against the duke jeopardy score to assess myocardium at risk

**DOI:** 10.1186/1532-429X-14-S1-O91

**Published:** 2012-02-01

**Authors:** Roy Jogiya, Kalpa De Silva, Geraint Morton, Simon Redwood, Sebastian Kozerke, Divaka Perera, Eike Nagel, Sven Plein

**Affiliations:** 1 Kings College London, London, UK; 2 LIGHT institute, Leeds, UK; 3 ETH, Zurich, Switzerland

## Summary

We demonstrate the feasibility of 3D myocardial perfusion CMR at 3 Tesla against invasive measures of myocardium at risk and ischaemic burden (Duke Jeopardy Score) in fifty-three patients for the detection of flow-limiting coronary artery disease and show good agrement between the techniques (Pearson r=0.82). This led to the novel use of this technique as a non-invasive method of detecting ischaemic burden and myocardium at risk for the purpose of serial studies, guiding revascularisation and risk stratification.

## Background

Three-dimensional (3D) myocardial perfusion cardiovascular magnetic resonance (CMR) overcomes the limited spatial coverage of conventional perfusion CMR methods. It permits whole heart coverage and can establish an estimation of myocardium at risk and ischaemic burden, which has not been validated as yet. For invasive estimation of ischaemic burden, semi-quantitative angiographic scores including the Duke Jeopardy Score have clinical legitimacy as the magnitude of myocardium at risk due to severe coronary stenosis is associated with an adverse prognosis. The Duke Jeopardy score combines assessment of stenosis severity and location.

## Objectives

To determine the association between myocardium at risk defined by the Duke Jeopardy Score and 3D CMR perfusion imaging.

## Methods

Fifty-three patients referred for angiography underwent rest and adenosine stress 3D myocardial perfusion CMR at 3Tesla (3D turbo gradient echo, flip angle 15, TR 2.0ms/TE 1.0ms, 12 slices of 5mm thickness, in-plane resolution 2.3x2.3mm2, 10 fold k-space and time k-t broad linear speed up technique acceleration with k-t principal component analysis).

Volume of myocardial hypoperfusion was calculated by a blinded observer using with GTVolume software (version 1.4.6, GyroTools, Zurich, Switzerland) with previously described quantitative methods based upon adjusting the signal intensity threshold >2 SDs below the signal of remote myocardium. Volume of hypoperfusion was calculated by summation of the contiguous slices.

Jeopardy score was calculated in each patient from the coronary angiograms to quantify the myocardium at risk. The coronary tree was divided into 6 segments of nearly equal myocardial perfusion (eg, left anterior descending artery, major diagonal branch, major septal branch, circumflex artery, major obtuse marginal branch artery, and posterior descending artery). A score of 2 for each significant lesion was given and an additional 2 points for each vessel distal to that lesion. Vessels were analysed by a cardiologist blinded to CMR and clinical details and assigned a score ranging from 0 (no Jeopardy) to 12 (maximum Jeopardy).

## Results

**Figure 1:**
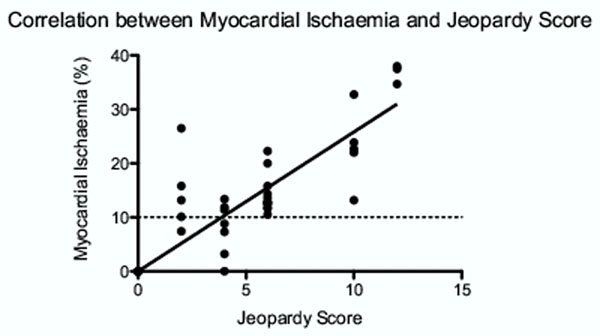
Strong correlation between invasive measures of disease severity and ischaemic burden (r=0.82). The dotted line represents the 10% threshold for which revascularisation may confer prognostic benefit over medical therapy alone

53 patients were scanned with 159 coronary vessels anaylsed. The mean percentage volume of hypoperfusion on 3D-CMR was 9.9% (±10.9). The mean Jeopardy Score was 4.0 (±3.9). The mean percentage volume of hypoperfusion for Jeopardy scores of 0, 6, 12 were 0, 13.1% and 36.7% respectively. Pearsons correlation coefficient showed a strong correlation (r=0.82, 95% CI 0.70 - 0.89) between the Jeopardy Score and volume of hypoperfusion on CMR (p<0.0001) (Figure 1).

## Conclusions

There is strong correlation between myocardium at risk by invasive indices and volume of inducible ischaemia by dynamic 3D CMR whole heart perfusion imaging. 3D CMR perfusion imaging offers a non-invasive alternative method of detecting ischaemic burden and myocardium at risk for the purpose of serial studies, guiding revascularisation and risk stratification.

## Funding

SP is funded by British Heart Foundation fellowship FS/10/62/28409.

SP/EN receives research grant support from Philips Healthcare.

